# α-Copaene is an attractant, synergistic with quercivorol, for improved detection of *Euwallacea* nr. *fornicatus* (Coleoptera: Curculionidae: Scolytinae)

**DOI:** 10.1371/journal.pone.0179416

**Published:** 2017-06-13

**Authors:** Paul E. Kendra, David Owens, Wayne S. Montgomery, Teresa I. Narvaez, Gary R. Bauchan, Elena Q. Schnell, Nurhayat Tabanca, Daniel Carrillo

**Affiliations:** 1 United States Department of Agriculture, Agricultural Research Service, Subtropical Horticulture Research Station, Miami, FL, United States of America; 2 United States Department of Agriculture, Agricultural Research Service, Beltsville Area Research Center, Electron and Confocal Microscopy Unit, Beltsville, MD, United States of America; 3 University of Florida, Tropical Research and Education Center, Homestead, FL, United States of America; Universidade Federal de Vicosa, BRAZIL

## Abstract

The tea shot-hole borer, *Euwallacea fornicatus* Eichhoff, is an ambrosia beetle endemic to Asia and a pest of commercial tea, *Camellia sinensis* (L.) Kuntze. Recently, a complex of species morphologically similar to *E*. *fornicatus* has been recognized, which includes new pests established in Israel and the USA, both in California and Florida. Collectively termed *E*. nr. *fornicatus*, these cryptic species carry symbiotic *Fusarium* spp. fungi, some of which cause dieback disease in susceptible hosts, which include avocado, *Persea americana* Miller. Due to the threat to this economically important crop, research was initiated to evaluate efficacy of kairomone-based lures for detection of the beetle in Florida (termed the Florida tea shot hole borer, FL-TSHB). A series of field tests were conducted in 2016 in commercial avocado groves known to have FL-TSHB at various population levels. All tests evaluated lures containing quercivorol (*p*-menth-2-en-1-ol) and α-copaene, presented separately and in combination; and one test evaluated effect of trap type on beetle captures. In addition, electroantennography (EAG) was used to quantify female olfactory responses to lure emissions. This study identified (-)-α-copaene as a new attractant for FL-TSHB, equivalent in efficacy to quercivorol (the standard lure for *Euwallacea* detection in the USA); however, the combination of lures captured significantly more FL-TSHB than either lure alone. This combination resulted in synergistic attraction at two field sites and additive attraction at a third site. Sticky panel traps captured more FL-TSHB than comparably-baited Lindgren funnel traps. Females engaged in host-seeking flight from 11:00 to 16:00 hr (EST), with peak numbers observed between 12:00 and 13:00 hr. EAG analyses confirmed olfactory chemoreception of both kairomones, with a higher response elicited with the combination of volatiles. Results indicate that detection of pest *E*. nr. *fornicatus* in Florida can be improved by using a two-component lure consisting of *p*-menth-2-en-1-ol and (-)-α-copaene.

## Introduction

Ambrosia beetles in the weevil subfamily Scolytinae (Coleoptera: Curculionidae) typically have a broad range of hosts, but target only stressed and dying trees. They are wood borers that excavate brood galleries in the host xylem and inoculate those galleries with spores of symbiotic fungi [1]. The resultant fungal gardens provide the sole nutritional source for both adult and larval beetles, but this colonization process also plays a vital ecological role by initiating and accelerating decomposition of dead wood. In general, ambrosia beetles seldom injure healthy trees within their endemic range; however, with an increase in global commerce, coupled with the challenge of detecting wood-boring insects at ports of entry [2], exotic ambrosia beetles pose an ever increasing threat to forest and agricultural ecosystems worldwide. Although benign in their native habitats, some species are acquiring pest status in newly established lands, as their fungal symbionts are now pathogenic in new (naïve) host trees [3].

*Euwallacea fornicatus* Eichhoff, the tea shot-hole borer (TSHB), is an ambrosia beetle native to Southeast Asia and a widespread pest of commercial tea, *Camellia sinensis* (L.) Kuntze [4]. In recent years, a complex of beetles morphologically indistinguishable from *E*. *fornicatus* have become established as pests of avocado, *Persea americana* Miller, and numerous other trees in Israel [5], in two regions of southern California [6–7], and in southern Florida [8–9]. Members of this cryptic group are referred to collectively as *E*. near *fornicatus*; all appear to be host generalists and carry combinations of symbiotic fungi in the genera *Fusarium*, *Graphium*, and *Acremonium* [9–11]. Some of those symbionts are phytopathogenic, causing localized lesions and branch dieback disease in infested trees [5–6, 9–10]. An initial phylogenetic analysis of this complex suggests that there are at least five genetically distinct beetle species and nine fungal symbionts, with *E*. nr. *fornicatus* sp. #1 (given the common name polyphagous shot hole borer, PSHB) found in Israel and California (originally established near Los Angeles), sp. #2 in Florida, sp. #3 in Australia, sp. #4 in Sri Lanka (presumably the original TSHB), and sp. #5 (the Kuroshio shot hole borer, KSHB) also in California, but south of the focal establishment of sp. #1, in the vicinity of San Diego [12]. More recently, a phylogeographic analysis suggests that populations of *E*. nr. *fornicatus* in Florida, Hawaii, Thailand, Australia, and Papua New Guinea all have a common source, and correspond to the TSHB [13].

Due to a lack of consensus regarding species status, and potential behavioral differences among *E*. nr. *fornicatus* populations that are geographically isolated, the species we studied will be referred to here as the Florida tea shot hole borer (FL-TSHB). This beetle was first detected in South Florida avocado groves in 2010, but the species was found in low numbers and not observed to cause serious damage at that time [8]. However, in the early spring of 2016, one grove was observed to have numerous infestations of FL-TSHB (~1500 trees) and considerable branch dieback [9]. In response to this change in pest status, research was initiated to gain a better understanding of the chemical ecology of the Florida species, including evaluations of kairomone-based lures. At present, lures containing quercivorol (*p*-menth-2-en-1-ol) are the standard for detection and monitoring of *E*. nr. *fornicatus* populations in both California and Florida [14]. However, in a field test targeting redbay ambrosia beetle (*Xyleborus glabratus* Eichhoff, the primary vector of laurel wilt) in Florida avocado groves, it was discovered that FL-TSHB was captured in traps baited with lures containing α-copaene [15], a sesquiterpene that functions as the principal host-location cue for dispersing female *X*. *glabratus* [16–17].

In this report, we summarize research conducted in several Florida avocado groves in 2016 to evaluate the efficacy of commercially available quercivorol and α-copaene lures, presented alone and in tandem, for attraction of in-flight female FL-TSHB. Field sites included a well-maintained grove that was free of laurel wilt, a grove affected by laurel wilt in which symptomatic trees were actively removed, and another grove with laurel wilt which contained numerous standing trees killed by laurel wilt. Thus, study sites varied considerably in their population levels of FL-TSHB. Data collection also documented the daily flight patterns of this pest beetle in southern Florida. In addition, quantitative electroantennographic analyses were performed to confirm chemoreception of these two semiochemicals, and to compare female olfactory responses to volatile emissions from the lure treatments evaluated in the field.

## Materials and methods

### Ethics statement

Field trials were conducted at three privately owned avocado groves in Miami-Dade County, FL, with permission of the landowners. Field studies targeted insect pests, and did not involve any protected or endangered species.

### Traps and lures

Commercial lures containing quercivorol and α-copaene were used in all field and laboratory experiments. Both lures consisted of clear plastic bubble dispensers, 29 mm in diameter, loaded with either 1-ml samples of quercivorol or 2-ml samples of α-copaene (Synergy Semiochemicals Corp., Burnaby, BC, Canada). Field tests were conducted using sticky panel traps, comparable to those used previously for evaluation of lures for *X*. *glabratus* [18]. Two white sticky cards (23 x 28 cm, Sentry wing trap bottoms; Great Lakes IPM, Vestaburg, MI, USA) were wired back-to-back along the upper edge, secured with a binder clip on the lower edge, and hung from tree limbs using s-shaped wire hooks. Lures were fastened to the wire hook just above the sticky cards, and the final assembly was topped with an inverted clear plastic plate (24 cm diam.) to provide a protective covering.

One field test also incorporated four-unit Lindgren funnel traps (BioQuip, Rancho Dominguez, CA, USA). With this trap design, lures were fastened to one of the side support arms midway between the second and third funnel. The collection cups were filled with 300 ml of an aqueous solution of 10% propylene glycol (Low-Tox antifreeze; Prestone, Danbury, CT, USA) to retain and preserve captured beetles.

### Field tests

Five field tests were conducted within three avocado groves during 2016, and each test included four treatments: a quercivorol lure, an α-copaene lure, a combination of quercivorol and α-copaene (2 separate lures secured side by side), and an unbaited control trap to assess random captures. Tests followed a randomized complete block design, with five replicate blocks (in all but one test; see below). Each block consisted of a row of traps hung ~1.5 m above ground [19], with a minimum spacing of 10 m between adjacent traps. Well shaded locations were chosen for placement of each trap.

Initial field trials were conducted in the grove where a high population of FL-TSHB was first detected in 2016 [9] (25^o^ 30’ 106” N, 80^o^ 29’ 372” W). This grove was well maintained, and three short tests (amounting to 8 weeks total trapping effort) were coordinated around the maintenance schedule of the grove manager. Field tests 1 and 2 were 2-week tests conducted from 24 February to 9 March, and from 4 to 18 May, respectively. Traps were checked three times a week in both tests. Field test 3 was a 4-week test, conducted from 30 June to 28 July, with traps checked once a week. At each sampling date, the sticky panels were collected, trap positions were rotated sequentially within each row (to minimize positional effects on beetle captures), and new sticky panels were hung.

Field test 4 was conducted in an avocado grove affected by laurel wilt, where diseased trees were cut down and destroyed by the grove manager (25^o^ 29’ 418” N, 80^o^ 28’ 593” W). This was an 8-week test, serviced weekly from 21 July to 15 September, following the general methods described above. However, since initial surveys indicated that FL-TSHB was present at very low levels in this grove, ten replicate blocks were deployed to improve sensitivity of beetle detection.

Field test 5 was conducted in another grove with laurel wilt (25^o^ 35’ 894” N, 80^o^ 27’ 847” W), but this site had many dead and symptomatic trees, with moderate levels of beetle infestation. This was a 6-week test checked weekly from 10 November to 22 December. Treatments were the same as those used in previous tests, but this test included an additional treatment consisting of a funnel trap baited with the combination lure. For both tests conducted in laurel wilt groves, blocks were set up in portions of the grove containing healthy (asymptomatic) trees which provided shade comparable to that found in the grove lacking laurel wilt.

Sticky panels collected from field tests were taken to the USDA-ARS laboratory (Miami, FL) and inspected under a dissecting microscope. For field tests 1 through 4, all species of Scolytinae were removed from the panels, soaked briefly in histological clear agent (Histo-clear II; National Diagnostics, Atlanta, GA, USA), counted, and identified based on morphological characters [20–21]. For field test 5, only specimens of FL-TSHB were counted.

### Determination of flight patterns

During the course of field test 1, data were collected on daily flight patterns of female FL-TSHB. On three sampling dates (4, 8, 9 March 2016), every trap (all treatments combined, n = 20) was inspected at 30-minute intervals to record the number of in-flight beetles captured throughout the day. Observations were initiated at 10:00 hours EST (when new sticky panels were hung) and continued until 16:30 hours (when captures ceased). For each collection day, the numbers caught were summed for each interval, and then converted to percentages of the total captures for that day.

### Electroantennography

Beetles used for EAG analyses were FL-TSHB females laboratory reared from infested avocado wood. Branches were cut from mature trees and held in plastic emergence chambers [44 gal (167 L) Brute® containers; Rubbermaid Commercial Products, Winchester, VA, USA]. To prevent condensation, ventilation holes were cut in the side and lid of each container and covered with fine mesh screening. To facilitate collection of newly emerged beetles, each container was fitted with a wide mouth 1 quart (0.95 L) mason jar (Ball Corp., Broomfield, CO, USA). This was done by cutting a circular hole (8.5 cm diameter) in the side of the container, and then gluing the metal ring lid in place, permitting easy removal of the jar. Moist tissue paper (Kimwipes, Kimberly-Clark, Roswell, GA, USA) was placed inside each jar, and beetles ≤ 24 hr post-emergence were collected each morning.

Test substrates consisted of commercial lures of quercivorol and α-copaene (as described above). The standard reference compound was ethanol (5 ml 95%; Pharmo-Aaper, Brookfield, CT, USA), which has been shown previously to elicit strong EAG responses in female ambrosia beetles [22]. Three sample bottles were prepared: quercivorol, α-copaene, and quercivorol + α-copaene. Substrates were placed into separate 250 ml glass bottles equipped with a lid containing a short thru-hull port (Swagelok, Solon, OH, USA) and silicone septum (Alltech, Deerfield, IL, USA). Sample bottles were then sealed and equilibrated for 2 hr at 24^°^C to allow for headspace saturation with volatiles.

**Fig 1:**
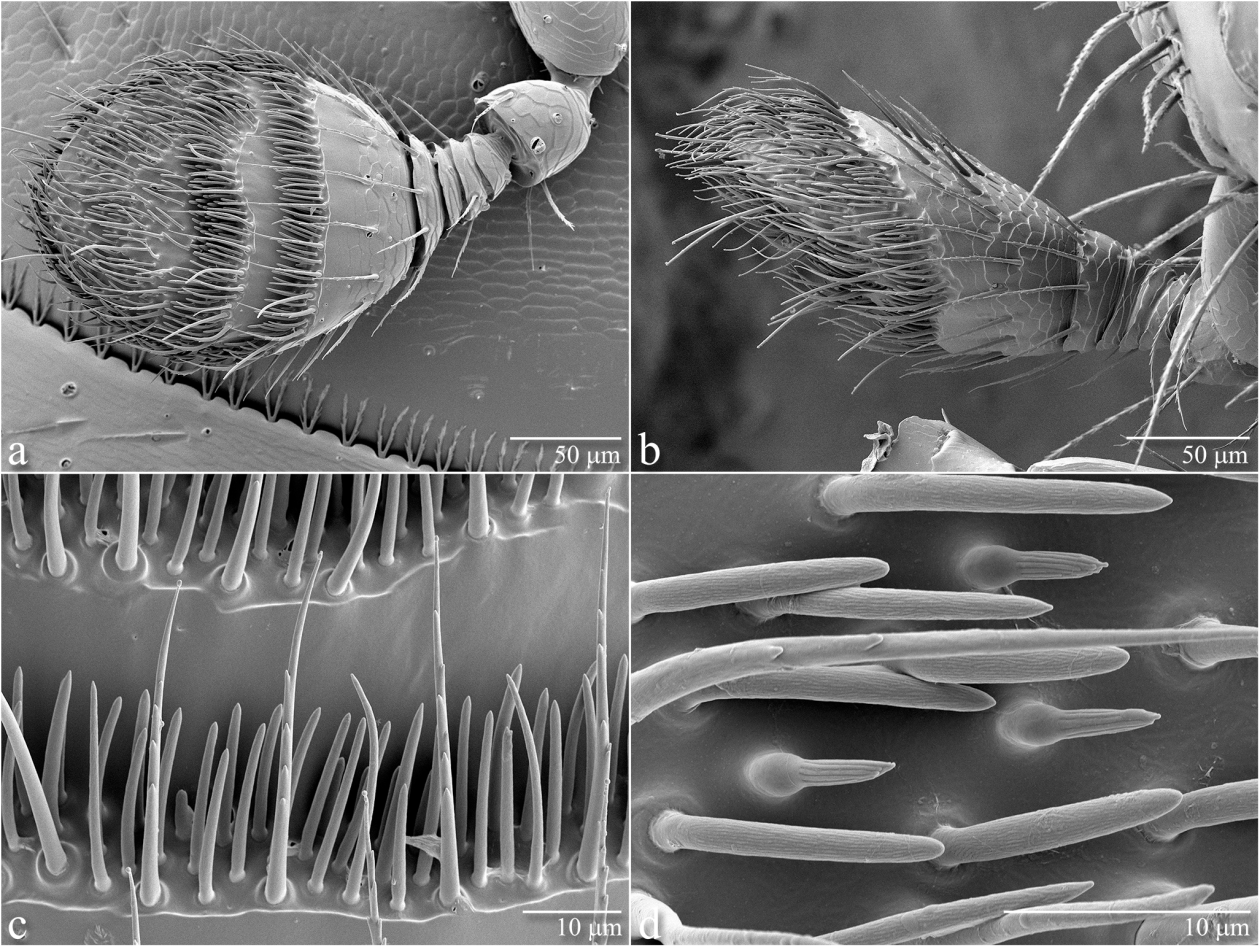
Scanning electron micrographs of antennae from female *Euwallacea* nr. *fornicatus*. (A) Ventral view of antenna, showing arrangement of sensilla in concentric rings on the apical end of antennal club (450 x magnification). (B) Lateral view of antenna, showing the diagonal elongation of the dorsal surface of the club and concentration of sensilla on the ventral surface (500 x magnification). (C) Portion of the lower concentric ring, showing two types of sensilla: long serrated sensilla trichoidea along the outer edge, and more numerous bluntly-ended sensilla basiconica throughout the interior (2000 x magnification). (D) Portion of the upper concentric ring, showing three types of sensilla: serrated sensilla trichoidea, blunt sensilla basiconica (with fine sculpturing), and shorter bulbous sensilla basiconica (with deep grooves) (5000 x magnification).

Peripheral olfactory responses were recorded with a Syntech EAG system and EAG 2000 software (Syntech Original Research Instruments, Hilversum, the Netherlands) using the general methods previously described for analyses with *Xyleborus* spp. [18, 22]. The antennal holder consisted of a gold-plated 2-pronged probe (Syntech Combi-Probe) which was modified by soldering a thin gold wire onto the different electrode [22]. Each preparation consisted of a single, excised antenna (mean length of 0.468 ± 0.008 mm, n = 20) and a small portion of the head capsule surrounding the socket. The basal portion was mounted onto the indifferent electrode using salt-free conductive gel (Spectra 360; Parker Laboratories, Fairfield, NJ, USA), and the flexible wire, coated with a thin layer of gel, was manipulated to make minimal contact with the apical antennal club. To facilitate appropriate EAG preparations, low-temperature scanning electron microscopy (LT-SEM) was used to visualize the antennal morphology of female FL-TSHB, using published methods [23–24]. LT-SEM images (Fig 1A–1D) indicated that the olfactory sensilla are not distributed evenly over the club, but primarily concentrated on the ventral surface in concentric rings. Therefore, antennae were positioned with the ventral surface facing upwards, and the gold wire electrode in contact with the dorsum of the club to prevent coating the sensilla with conductive gel.

Antennal preparations were placed under a stream (400 ml/min) of humidified air, purified with activated charcoal granules (grain size 1–2 mm). Using gas-tight syringes (SGE Analytical Science, Victoria, Australia), samples of saturated vapor were withdrawn from test bottles, injected into the airstream, and delivered to the antennae. In each recording session, the antenna was presented with samples in the following order: the ethanol standard (2 ml dose), samples of volatiles emitted by test lures, a negative control (clean air injection equal in volume to the test samples), and a final ethanol standard. Injections were delivered at 2 min intervals, with a clean air flush in between, to minimize antennal adaptation.

Test responses were recorded initially in millivolts (peak height of depolarization) and then normalized to percentages relative to the response obtained with the ethanol standard. Normalization corrects for time-dependent decline in antennal performance, and also allows for comparison of relative EAG responses obtained with different substrates [25–27] and with different cohorts of insects [28–29]. Any response obtained with the negative control was subtracted from the normalized response, and the final corrected values were used to make comparisons among test samples.

Two EAG experiments were conducted with lure volatiles. The first experiment quantified dose-dependent responses to volatiles emitted from the quercivorol lure, using five volumetric doses ranging from 0.5 to 4.0 ml. Based on the results of this initial test, the second EAG experiment used fixed doses to compare olfactory responses to volatiles from the three lure treatments. Responses were recorded from 10 females to construct the quercivorol dose-response curve, and from 15 females in the comparative EAG experiment.

After the experiments were concluded, gas chromatographic (GC) analysis was performed to quantify headspace volatiles from the EAG sample bottles. An internal standard, 600 μl hexadecane (99%, Sigma Aldrich, St. Louis, MO, USA), was introduced into each bottle using a 1 ml syringe (SGE Analytical Science), and bottles were left undisturbed for >48 hr to allow volatiles to equilibrate prior to sampling. Headspace samples (10 μl each, 20 replicates per sample bottle) were collected with a 25 μl syringe (Pressure Lok; VICI Precision Sampling, Baton Rouge, LA, USA) and analyzed using a TRACE GC Model 2000 (Thermo Electron North America LLC, West Palm Beach, FL, USA) fitted with a J&W DB-5MS column, 25m x 0.25mm x 0.25 μm (Agilent Technologies, Santa Clara, CA, USA). The split/splitless injector was set at 225^°^C; helium was the carrier gas set at a constant flow of 0.8 ml/min. The flame ionization detector (FID) was set at 250^°^C. The oven was set up with a temperature ramp program, starting at 50^°^C and increasing at 15^°^C/min to 130^°^C, then at 10^°^C/min to 220^°^C and holding at that temperature for 2 min.

### Statistical analysis

Regression analysis was used to describe the relationship between substrate dose and response of olfactory receptors to volatiles emitted from the quercivorol lure (first EAG experiment), and also to document the relationship between time of day and the number of female FL-TSHB observed in flight. One-way analysis of variance (ANOVA) was used to test the effect of treatment on attraction (field tests) and olfactory response (comparative EAG experiment); significant ANOVAs were then followed by mean separation with the Tukey HSD test. To evaluate potential synergism between lures, analysis by *t*-test was used to compare the field captures obtained with the combination lure to the summed captures obtained with individual α-copaene and quercivorol lures. In addition, analysis by *t*-test was used to compare EAG responses obtained with adjacent doses of quercivorol. When necessary, data were square root (*x* + 0.5)-transformed to stabilize variance prior to analysis. All analyses were performed using Systat Software [30]. Results are presented as mean ± SEM; probability was considered significant at a critical level of α = 0.05.

## Results

### Field tests

**Fig 2:**
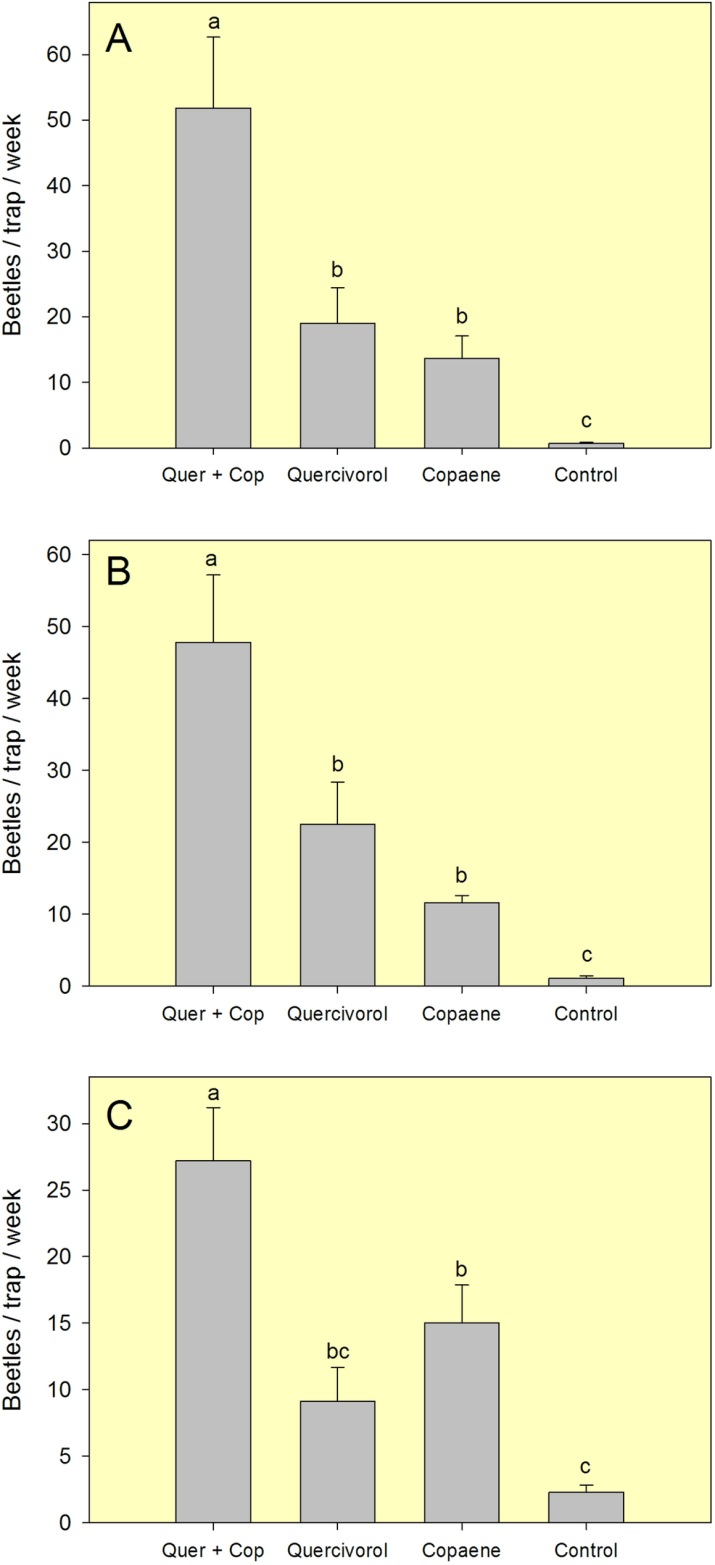
Mean (±SEM) captures of female *Euwallacea* nr. *fornicatus* in field tests conducted in a well maintained avocado grove in Miami-Dade County, FL. (A) 2-week test in Feb-Mar 2016. (B) 2-week test in May 2016. (C) 4-week test in Jun-Jul 2016. Lure treatments consisted of a quercivorol bubble lure and an α-copaene bubble lure, deployed separately and in combination in sticky panel traps. The control treatment consisted of an unbaited sticky trap. Bars topped with the same letter are not significantly different (Tukey HSD means separation, *P* < 0.05).

In field test 1 (Fig 2A, S1 Table), field test 2 (Fig 2B, S2 Table), and field test 3 (Fig 2C, S3 Table)–the trials conducted in an avocado grove without laurel wilt–there were differences in mean capture of FL-TSHB among the four treatments evaluated (*F* = 17.018, df = 3,16, *P* < 0.001; *F* = 12.970, df = 3,16, *P* < 0.001; *F* = 9.398, df = 3,16, *P* < 0.001, respectively). In all three tests, highest captures were obtained with the combination of quercivorol and α-copaene lures, intermediate captures with single lures of either quercivorol or α-copaene, and fewest captures with the unbaited control trap. The combination of α-copaene and quercivorol resulted in an additive effect on captures of FL-TSHB. Captures of non-target Scolytinae were few. In addition to FL-TSHB, ten other species were captured, with *Ambrosiodmus devexulus* (Wood) being the species most commonly observed (Table 1). With the collective captures from this field site, FL-TSHB comprised 89.0, 95.8, and 96.0% of the scolytine beetles intercepted in traps baited with α-copaene, quercivorol, and the combination lure, respectively.

**Table 1:** Summed captures of Scolytinae (Coleoptera: Curculionidae) from three field tests conducted in a well-managed avocado grove in Miami-Dade County, FL (8 wk total trapping effort, Feb-Jul 2016).

Species		Quercivorol + α-Copaene	Quercivorol	α-Copaene	Unbaited Control
Tribe Xyleborini					
	*Ambrosiodmus devexulus* (Wood)	34	8	31	4
	*Ambrosiodmus lecontei* Hopkins		1		1
	*Euwallacea nr*. *fornicatus*	1514	620	573	52
	*Premnobius cavipennis* Eichhoff			1	
	*Theoborus ricini* (Eggers)	7	4	13	8
	*Xyleborinus andrewesi* (Blandford)	1		1	
	*Xyleborinus gracilis* Eichhoff	8	1	5	
	*Xyleborinus saxesenii* (Ratzeburg)	1	1	2	1
	*Xyleborus bispinatus* Eichhoff		1	1	
	*Xylosandrus crassiusculus* (Motschulsky)	6	2		
Tribe Cryphalini					
	*Hypothenemus* spp.	6	9	17	3

**Fig 3:**
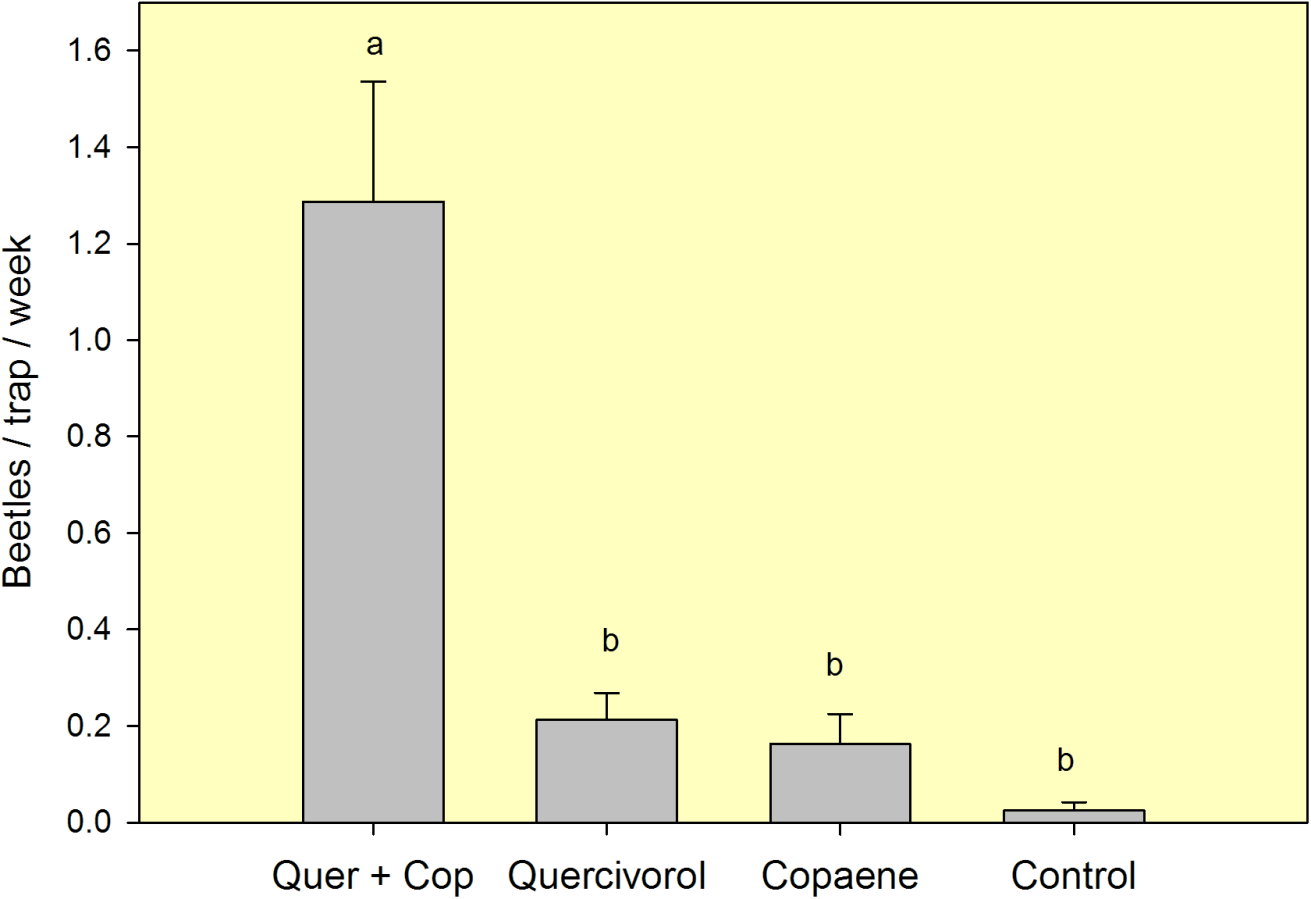
Mean (±SEM) captures of female *Euwallacea* nr. *fornicatus* in an 8-week field test (Jul-Sep 2016) conducted in a managed avocado grove with laurel wilt in Miami-Dade County, FL. Lure treatments consisted of a quercivorol bubble lure and an α-copaene bubble lure, deployed separately and in combination in sticky panel traps. The control treatment consisted of an unbaited sticky trap. Bars topped with the same letter are not significantly different (Tukey HSD means separation, *P* < 0.05).

In field test 4 (Fig 3, S4 Table), conducted in the managed grove with laurel wilt, there were also differences in mean captures of FL-TSHB among lure treatments (*F* = 24.494, df = 3,36, *P* < 0.001). As with tests 1–3, captures were highest with the combination lure. However, at this site with very low levels of FL-TSHB, captures with the single lures were not statistically greater than those obtained with the unbaited control. Consequently, mean captures with the combination lure were significantly greater than the summed captures from the two traps baited with single lures (*t* = 3.736, df = 18, *P* = 0.002), suggesting synergistic attraction. In contrast to the previous field site, captures of non-target Scolytinae were numerous; thirteen other species were encountered, with the most common including *Xyleborus bispinatus* Eichhoff, *Ambrosiodmus devexulus*, and *Xyleborus volvulus* Eichhoff (Table 2). Traps baited with α-copaene and the combination lure also caught several specimens of *X*. *glabratus*. FL-TSHB was the second most abundant species, but comprised only 7.7, 11.3, and 35.4% of the scolytine beetles caught with α-copaene, quercivorol, and the combination lure, respectively.

**Table 2:** Summed captures of Scolytinae (Coleoptera: Curculionidae) from an 8-wk field test conducted in an avocado grove affected by laurel wilt in Miami-Dade County, FL (Jul-Sep 2016).

Species		Quercivorol + α-Copaene	Quercivorol	α-Copaene	Unbaited Control
Tribe Xyleborini					
	*Ambrosiodmus devexulus* (Wood)	45	4	29	5
	*Ambrosiodmus lecontei* Hopkins		1	1	
	*Euwallacea nr*. *fornicatus*	101	16	13	2
	*Theoborus ricini* (Eggers)			1	
	*Xyleborinus andrewesi* (Blandford)	3	1	2	
	*Xyleborinus saxesenii* (Ratzeburg)	1	3	3	2
	*Xyleborus affinis* Eichhoff	2	8	7	6
	*Xyleborus bispinatus* Eichhoff	89	79	78	49
	*Xyleborus ferrugineus* (F.)	1	3	3	4
	*Xyleborus glabratus* Eichhoff	2		2	
	*Xyleborus volvulus* Eichhoff	21	15	14	5
	*Xylosandrus compactus* (Eichhoff)	2	2	2	1
	*Xylosandrus crassiusculus* (Motschulsky)	9	3	1	
Tribe Cryphalini					
	*Hypothenemus* spp.	9	7	13	2

**Fig 4:**
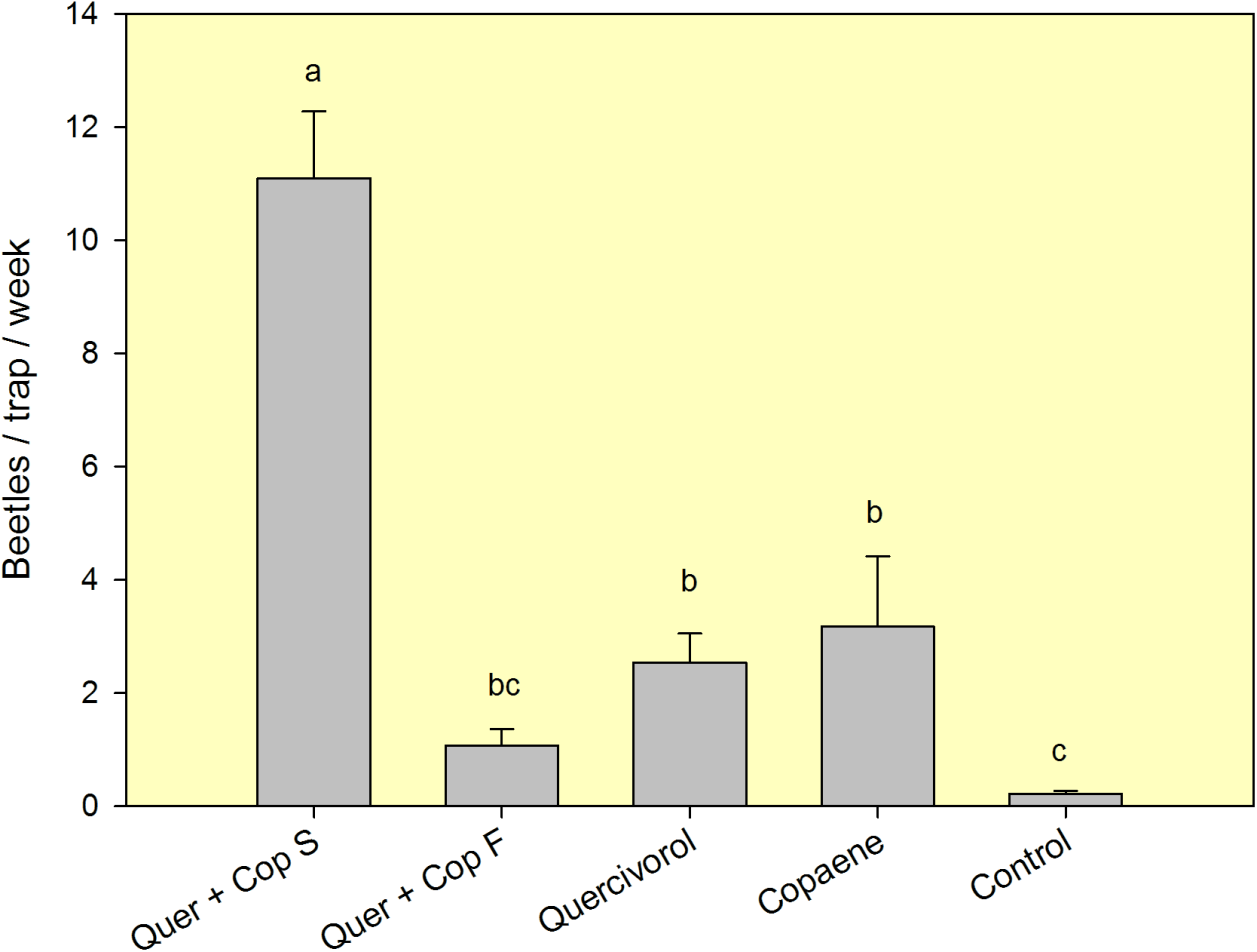
Mean (±SEM) captures of female *Euwallacea* nr. *fornicatus* in a 6-week field test (Nov-Dec 2016) conducted in an unmanaged avocado grove with laurel wilt in Miami-Dade County, FL. Lure treatments consisted of a quercivorol bubble lure and an α-copaene bubble lure, deployed separately in sticky panel traps, and deployed in tandem in a sticky panel trap (S) and a Lindgren funnel trap (F). The control treatment consisted of an unbaited sticky trap. Bars topped with the same letter are not significantly different (Tukey HSD means separation, *P* < 0.05).

Consistent with previous results, mean captures of FL-TSHB in field test 5 (Fig 4, S5 Table) were also significantly different among treatments (*F* = 28.222, df = 4,20, *P* < 0.001). Highest captures were obtained with sticky traps baited with the combination lure, intermediate captures with single quercivorol or α-copaene lures, and lowest captures with the unbaited control. Lindgren funnel traps baited with the combination lure captured significantly fewer beetles than equally baited sticky traps, and captures with baited funnel traps were no different than those obtained with unbaited sticky panel controls. As in the other grove with laurel wilt, synergistic attraction of FL-TSHB was observed with the combination lure; mean captures with the combination lure deployed in sticky traps were significantly higher than the summed captures from the two single-baited traps (*t* = 3.318, df = 8, *P* = 0.011). At this site with laurel wilt, a single *X*. *glabratus* was captured in a trap baited with the combination lure, and several *X*. *glabratus* were captured with α-copaene lures in an initial test conducted within this avocado grove in 2015 [15].

### Temporal patterns in host-seeking flight

**Fig 5:**
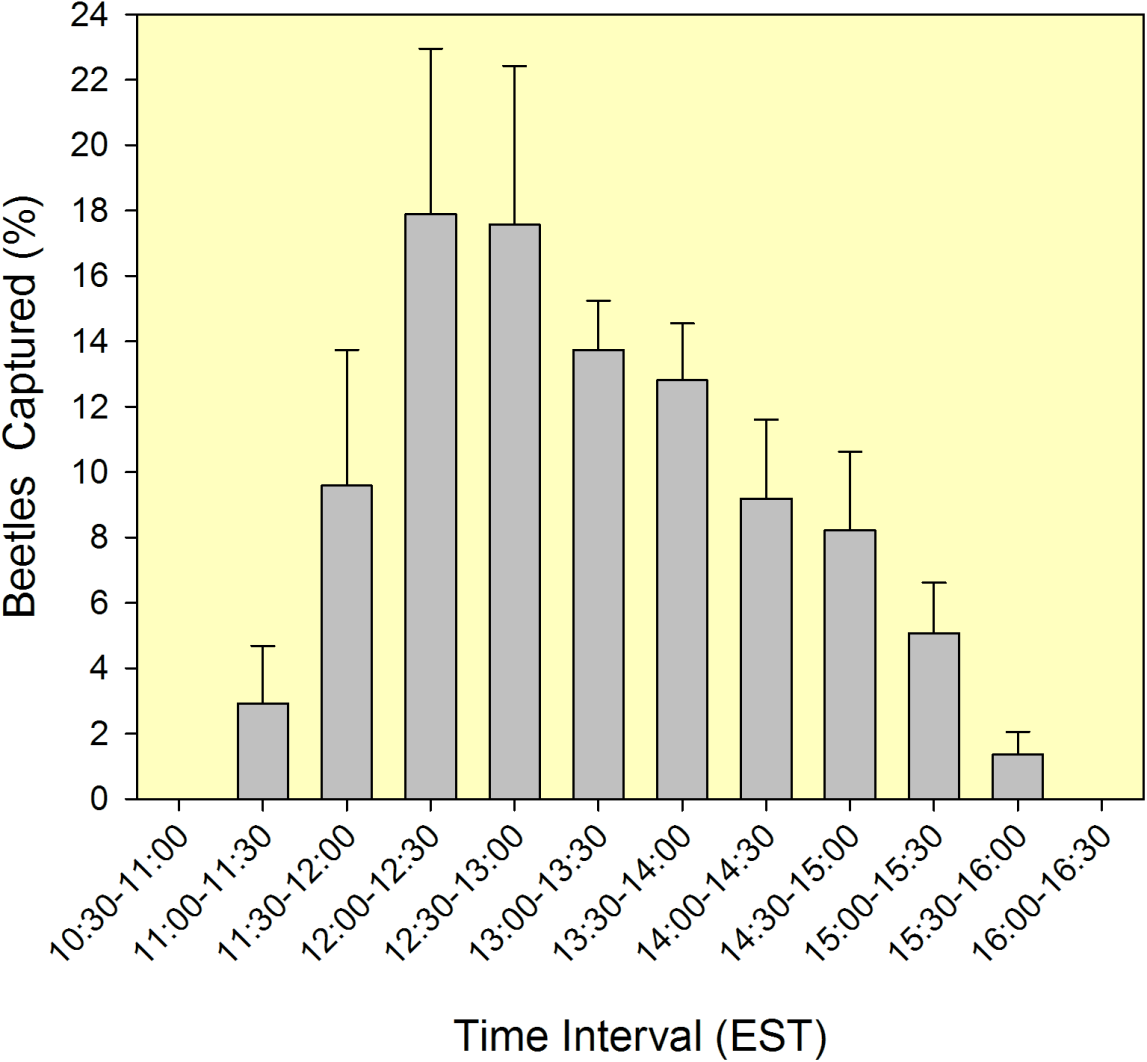
Diel patterns in host-seeking flight of female *Euwallacea* nr. *fornicatus* in a southern Florida avocado grove (Miami-Dade County). Graph summarizes captures (mean ± SEM) of beetles in sticky panel traps (all traps deployed in field test 1, n = 20, conducted in Feb-Mar 2016) for each 30-minute interval from 10:30–16:30 hours (Eastern Standard Time).

Females of FL-TSHB were observed in flight as early as 11:00 hours EST, with peak flight from 12:00 to 13:00 hours (Fig 5, S6 Table). Numbers gradually declined throughout the afternoon, with no captures observed after 16:00 hours. This pattern was best fit by regression with a Gaussian peak model (*y* = 16.95e^-0.5[(*x*-12.77)/1.19]2^, *R*^2^ = 0.882).

### Electroantennography

**Fig 6:**
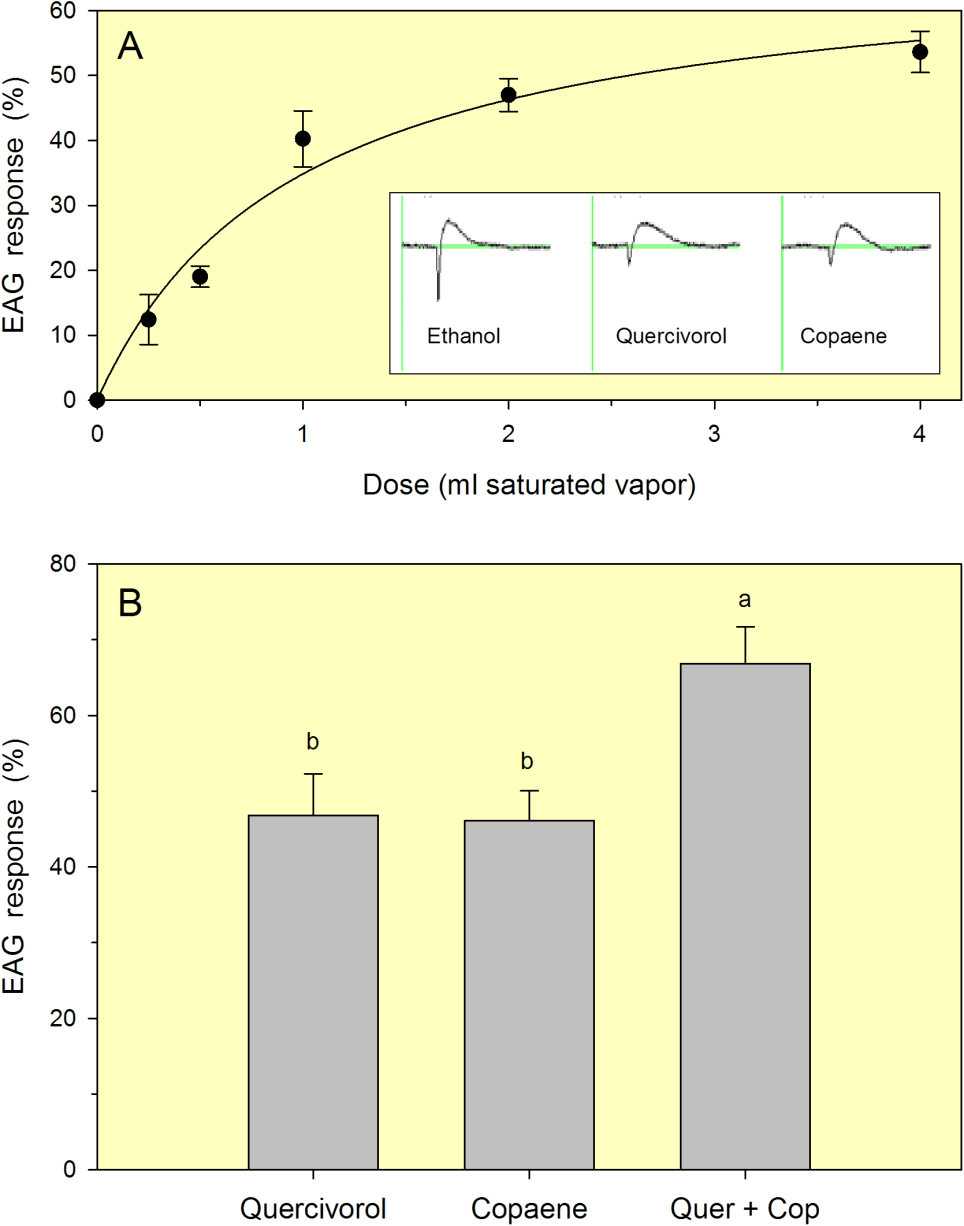
Mean (±SEM) electroantennogram responses of female *Euwallacea* nr. *fornicatus* to volatiles emitted from commercial lures containing quercivorol and α-copaene. (A) Dose-response curve obtained with a series of volumetric doses of volatiles from the quercivorol lure; curve generated with a hyperbolic regression model (see text). Insert depicts representative electroantennogram recordings obtained with single excised antennae to chemicals used in this study. (B) Comparative electroantennogram responses (mean ±SEM) to fixed 2-ml doses of volatiles emitted from a single quercivorol lure, a single α-copaene lure, and a combination of the two lures. All responses are expressed as normalized percentages relative to the standard reference compound (ethanol, 2-ml saturated vapor). Bars topped with the same letter are not significantly different (Tukey HSD means separation, *P* < 0.05).

The EAG techniques developed for *Xyleborus* spp. [22] worked well for quantification of olfactory responses from *Euwallacea* antennae, with representative electroantennograms shown in Fig 6A. The relationship between dose of volatiles from the quercivorol lure and the amplitude of EAG response was best fit by regression with a hyperbolic model [*y* = 68.96*x*/(0.98 + *x*), *R*^2^ = 0.976; Fig 6A, S7 Table]. Amplitude increased with dosage up through the 1-ml dose (mean EAG response to the 1-ml dose was greater than response to the 0.5-ml dose; *t* = -4.62, df = 14, *P* < 0.001), and then began to level off. EAG responses did not increase significantly as dose doubled from 1 to 2 ml (*t* = -1.35, df = 14, *P* = 0.198) or from 2 to 4 ml (*t* = -1.63, df = 14, *P* = 0.126). This observed plateau in EAG response suggested that the dose range evaluated was sufficient to elicit maximal antennal response (saturation of olfactory receptors), and a fixed 2-ml dose was chosen for the comparative EAG experiment.

There were significant differences in antennal responses to volatile emissions from the three lure treatments (*F* = 5.997; df = 2,33; *P* = 0.006; Fig 6B, S8 Table). Mean EAG response elicited with the combination of quercivorol and α-copaene was significantly greater than the responses observed with either lure alone. Mean antennal responses were comparable when volatiles from the two lures were presented separately.

Quantification of headspace volatiles from the EAG sample bottles (S9 Table) indicated that saturated vapor from the α-copaene lure contained 7.92 ± 0.44 mg/ml of α-copaene. Saturated vapor from the quercivorol lure contained two components: a dominant isomer (*cis*-*p*-menth-2-en-1-ol, 86.6%) and a secondary isomer (*trans*-*p*-menth-2-en-1-ol, 13.4%), quantified at 4.06 ± 0.42 and 0.63 ± 0.06 mg/ml, respectively. Analysis of headspace from the bottle containing both lures indicated a composition of 47.4% α-copaene, 44.3% *cis*-*p*-menth-2-en-1-ol, and 8.3% *trans*-*p*-menth-2-en-1-ol, quantified at 2.85 ± 0.30, 2.66 ± 0.29, and 0.50 ± 0.06 mg/ml, respectively.

## Discussion

*Fusarium* dieback poses a substantial economic threat to avocado production worldwide. Pest *Euwallacea* populations established in California (PSHB, KSHB) and in Israel (PSHB) currently impact the avocado industries in those production areas [6, 31], where the commodity values for the 2015–2016 season were 274.4 million USD [32] and 39.9 million USD [33], respectively. An initial survey in Florida, where the 2016 market value was 20.6 million USD [32], revealed that FL-TSHB is already prevalent throughout the commercial avocado groves in Miami-Dade County, albeit at sub-economic thresholds in most locations [9]; however, population levels are likely to increase over time due to the large monoculture of suitable hosts. Moreover, these exotic pests can be highly polyphagous, thereby severely threatening native ecosystems; the PSHB and KSHB in California have been reported to attack more than 300 host species within 58 plant families [7]. Clearly, there is a critical need for integrated pest management of these invasive *Euwallacea* spp., and the results presented in this report identify α-copaene as a new attractant for FL-TSHB, and the combination of α-copaene and quercivorol as an improved lure for early pest detection. When deployed in the avocado grove unaffected by laurel wilt, the two-component lure was highly specific for FL-TSHB, with non-target Scolytinae comprising only 4% of the captures.

Although *Fusarium* dieback is a newly emerging concern in Florida, the avocado industry in that state is already experiencing significant losses due to another vector-borne disease, laurel wilt. Unlike dieback disease, where *Fusarium* migration is minimal beyond the beetle galleries, the laurel wilt pathogen, *Raffaelea lauricola* T. C. Harrington, Fraedrich & Aghayeva [34], is transported systemically throughout the host xylem system [35]. Presence of this foreign fungus triggers a cascade of host defensive responses [36] which ultimately causes a vascular disease lethal to avocado and other woody Lauraceae [37–41]. Although *R*. *lauricola* was introduced into the USA as a symbiont of the Asian redbay ambrosia beetle, *X*. *glabratus* [42], there has been an unprecedented lateral transfer of this mycopathogen to native ambrosia beetles that co-breed in infected hosts [43]. In 2011 it was noted that there are potentially many additional vectors, as numerous ambrosia beetles in Florida are attracted to, and colonize, the same substrates as *X*. *glabratus* [44]. As of 2016, *R*. *lauricola* has been recovered from the mycangia (spore storage organs) of ten species of ambrosia beetle in Florida [45]. Although *X*. *glabratus* is still the primary and most efficient vector, other species have been shown to function as secondary vectors under greenhouse conditions [43]. The combination of multiple vectors, movement of infested wood, and a lack of cost-effective controls for both the vector(s) and pathogen poses a formidable challenge for management of laurel wilt [41]. From a single introduction point in Georgia in 2002 [20], *X*. *glabratus* and laurel wilt have spread quickly throughout the southeastern USA; they are now established in nine states, as far west as Texas [46]. With future spread, laurel wilt could impact avocado production in California and in Mexico, the world's largest supplier, with 176,000 ha of avocado grown and where production is valued at 1.2 billion USD [47–48]. Furthermore, the KSHB from southern California has recently invaded Mexico (outside Tijuana). In response to the imminent threat posed by both *Fusarium* dieback and laurel wilt, Mexico in 2013 began surveying commercial avocado groves and high risk areas (ports, airports, and international borders) using Lindgren traps baited with quercivorol and serviced weekly [48], and hosted the Academic and Technical Workshop on *Xyleborus glabratus* and *Euwallacea* sp. at the Instituto de Ecología, in Xalapa, Veracruz in November 2014 [49].

Although the fungal pathogens and disease expression are very different with *Fusarium* dieback and laurel wilt, there are a number of characteristics common to the two beetle vectors. First, the two beetles are atypical in terms of their selection of breeding substrates. Most ambrosia beetles seek out trees that are stressed, weakened, and dying, but both *X*. *glabratus* and *E*. nr. *fornicatus* spp. can function ecologically as primary colonizers, readily attacking live (apparently healthy) hosts [6, 50]. With *X*. *glabratus*, it has been reported that initial attacks on healthy trees may not be successful for colonization, but sufficient to introduce *R*. *lauricola*; once trees are infected and symptomatic for laurel wilt, subsequent attacks are more likely to result in colonization and reproduction [42]. Research is needed to determine if this applies to *E*. nr. *fornicatus* spp. as well.

Second, the two species differ from the majority of ambrosia beetles in terms of their chemical ecology and utilization of host-location cues. Neither species is strongly attracted to ethanol [14, 18, 50], a volatile indicative of tree stress and/or decay, which is the standard lure for general detection of ambrosia beetles [51]. Rather, they appear to rely more on kairomones released by healthy host trees. With *X*. *glabratus*, the current hypothesis is that dispersing females are attracted to a characteristic bouquet of the Lauraceae [52–54]. The primary host-orientation cue has been shown to be (-)-α-copaene, the enantiomer found in the commercial α-copaene lure [16–18]. However, a comparative study of nine lauraceous species indicated that emissions of several other sesquiterpenes are also correlated with field captures of *X*. *glabratus*, including α-cubebene, α-humulene, and calamenene [38]. The monoterpene ether eucalyptol (1,8 cineole) [55] and even host leaf volatiles [56] may contribute to that signature bouquet as well. Through serendipity, it was discovered that FL-TSHB is also attracted to the α-copaene lure. Since both attraction and EAG responses to α-copaene were equivalent to those of quercivorol (the only other strong attractant known for *Euwallacea*), α-copaene may indeed be a primary host cue for FL-TSHB, but additional work is needed to identify the reproductive hosts of FL-TSHB, and to evaluate potential kairomones emitted from those hosts. At the study site used for field tests 1–3, plantings included multiple cultivars of avocado; however, a single variety (cv. 'Donnie') was preferentially attacked by FL-TSHB. This anecdotal evidence suggests that differences in host kairomone emissions (genetically based) affect attraction of host-seeking FL-TSHB. Analysis of the terpenoid content of the 'Donnie' cultivar is warranted, and further lure improvement may be possible if additional attractants are identified. This information may also facilitate breeding programs for avocado cultivars less susceptible to attack by FL-TSHB. It is also worth noting that the α-copaene lure is the only commercially available lure that has been able to detect *X*. *glabratus* at very low population levels in Florida avocado groves [15] (and the current study), and now has been shown to serve a dual purpose for detection of that pest and the FL-TSHB.

Third, both beetle species are attracted to volatiles from their fungal symbionts (i.e. food-based odors). With *X*. *glabratus*, binary choice tests demonstrated attraction of females to cultures containing *R*. *lauricola* [57], and subsequent field tests showed that fungal volatiles were synergistic with (host-based) essential oil lures to increase captures of in-flight females [58]. Comparable results were observed in our field tests for FL-TSHB. Lures containing quercivorol (a fungal attractant, as reported by the lure manufacturer), in combination with the host-based α-copaene lures, resulted in either an additive effect (at high population levels) or a synergistic effect (at lower population levels) on captures of FL-TSHB. However, the manufacturer indicated that the commercial lures contain *p*-menth-2-en-1-ol, which, due to two chiral centers, is actually a mixture of four isomers. Our GC analysis of the EAG sample headspace, performed on a conventional column, separated the *cis*- and *trans-* isomers in a ratio of 87:13, but could not distinguish between diastereomers (separation on a chiral column is required to resolve all four isomers). To be precise, only one of the *cis-* molecules, (1*S*, 4*R*)-*p*-menth-2-en-1-ol, was assigned the common name quercivorol by researchers working on *Platypus quercivorus* Murayama (Coleoptera: Curculionidae: Platypodinae) in Japan [59]. Further studies are needed to determine if FL-TSHB is indeed attracted to quercivorol or to one (or a combination) of the other isomers. Until that time, it is more accurate to state that FL-TSHB is attracted to *p*-menth-2-en-1-ol.

Last, the flight window of both beetle species is atypical for Scolytinae within the tribe Xyleborini. Most ambrosia beetles (at least the fauna in Florida) engage in host-seeking flight during the early evening, with peak numbers observed near sunset and flight continuing after dark [22, 60]. In fact, many species in Florida are attracted to ultraviolet light traps [61]. In contrast, females of *X*. *glabratus* initiate flight in the late afternoon and flight ceases before sunset [22], and females of FL-TSHB fly even earlier, with peak flight occurring during the early afternoon hours. Since both species have diurnal flight patterns, it is likely that they incorporate visual cues in their host-location repertoire. For *X*. *glabratus*, it has been shown that stem silhouette diameter functions as a host visual cue, but only when presented concurrently with appropriate host odors [62]. Comparable experiments are needed to evaluate visual cues for FL-TSHB, as these may potentially improve trapping systems for this pest. It is thought that the black nested funnels of the Lindgren trap provide an attractive visual cue (tree trunk silhouette) for ambrosia beetles, but this was not supported by our results. White sticky panels captured more FL-TSHB than Lindgren funnel traps, despite both traps being baited with the combination lure. A similar pattern was observed with *X*. *glabratus* when equally-baited funnel traps and sticky panels were compared [63].

## Conclusions

Adventive ambrosia beetles in the *Euwallacea* nr. *fornicatus* cryptic species complex constitute a global threat to both agricultural and natural ecosystems. Early pest detection is critical for development of integrated pest management programs to mitigate the impact of these species. The current research identifies (-)-α-copaene as a new attractant for *Euwallacea* nr. *fornicatus* in Florida, and a multi-component lure containing (-)-α-copaene and *p*-menth-2-en-1-ol as a new tool for more sensitive pest detection. Due to similarities between the FL-TSHB and the redbay ambrosia beetle, our knowledge pertaining to the latter species may provide insight into the biology and ecology of the FL-TSHB, and help direct future research efforts on this new invasive pest.

## Supporting information

S1 Table Captures of female *Euwallacea* nr. *fornicatus* in field test 1. (XLSX)

S2 Table Captures of female *Euwallacea* nr. *fornicatus* in field test 2. (XLSX)

S3 Table Captures of female *Euwallacea* nr. *fornicatus* in field test 3. (XLSX)

S4 Table Captures of female *Euwallacea* nr. *fornicatus* in field test 4. (XLSX)

S5 Table Captures of female *Euwallacea* nr. *fornicatus* in field test 5. (XLSX)

S6 Table Temporal flight patterns of female *Euwallacea* nr. *fornicatus*. (XLSX)

S7 Table EAG dose-response to volatiles from quercivorol lure. (XLSX)

S8 Table Comparative EAG responses to fixed doses of volatiles from lure treatments. (XLSX)

S9 Table Quantification of volatiles in EAG sample bottles. (XLSX)
